# Average absorbed breast dose in mammography: a new possible dose index matching the requirements of the European Directive 2013/59/EURATOM

**DOI:** 10.1186/s41747-017-0026-1

**Published:** 2017-12-28

**Authors:** Antonio C. Traino, Chiara Sottocornola, Patrizio Barca, Carolina Marini, Giacomo Aringhieri, Davide Caramella, M. Evelina Fantacci

**Affiliations:** 1 U.O.Fisica Sanitaria, Azienda Ospedaliero-Universitaria Pisana, via Roma n.67, 56122 Pisa, Italy; 2 Dipartimento di Fisica “E.Fermi”, Università di Pisa, L.go B.Pontecorvo n.3, 56127 Pisa, Italy; 3 S.D.Radiologia Senologica, Azienda Ospedaliero-Universitaria Pisana, via Roma n.67, 56122 Pisa, Italy; 4 U.O.Radiodiagnostica 1, Azienda Ospedaliero-Universitaria Pisana e Università di Pisa, via Paradisa n.2, 56100 Pisa, Italy

**Keywords:** Average glandular dose, Breast absorbed dose, Dosimetry, Mammography, Ionising radiation dose index

## Abstract

**Background:**

The new European Directive 2013/59/EURATOM requires that patients are informed about the risk associated with ionising radiation and that detailed information on patient exposure is included in the radiological report. This implies a revision of the routinely used dose indexes to obtain quantities related to individual exposure evaluable from acquisition parameters. Here we propose a new mammography dose index consistent with the average glandular dose (*AGD*).

**Methods:**

An equation has been developed for calculating the average absorbed breast dose (*2ABD*). It depends on incident air kerma *k*_*a,i*_ and on energy absorption coefficient *μ*_en_; *k*_*a,i*_ can be calculated for each anode-filter combination, based on *kVp*, *mAs*, the yield of the tube used *Y*_*tb*_, and the breast thickness *d*; *μ*_*en*_ depends on *kVp* and has been evaluated for each anode-filter combination. *2ABD* has been compared to *AGD* evaluated by Dance or Wu methods, which represent the reference standards, for 20 patients of our university hospital.

**Results:**

The incident air kerma *k*_*a,i*_, calculated as a function of *kVp*, *mAs*, *Y*_*tb*_ and *d*, was in good agreement with the same quantity directly measured: the relative uncertainty is < 0.10. The results of the comparison between *2ABD* and *AGD* evaluated by both Dance and Wu methods appear to be consistent within the uncertainties.

**Conclusions:**

*2ABD* is easily evaluable for each mammogram from the acquisition parameters. It can be proposed as a new suitable dose index, consistent with *AGD*, matching the requirements of the 2013 European Directive.

## Key points

- A new dosimetric quantity (average absorbed breast dose [*2ABD*]) is presented
- 2ABD is a physics quantity directly measurable
- 2ABD is consistent with average glandular dose (reference metrics)
- 2ABD can be easily calculated and thus reported in the mammographic radiological report
- 2ABD matches the requirements of the new European Directive 2013/59/Euratom

## Background

Breast cancer is the most common female cancer worldwide and the second most common cancer overall, with more than 1,700,000 new cases diagnosed in 2012, equal to 25% of female cases and 12% of the total [1].

Mammography is considered the most effective imaging technique for the early detection and diagnosis of breast cancer: it uses low-energy ionising radiation to produce a mammogram that is a radiographic two-dimensional projection of the breast. However, the breast is a highly radiosensitive organ: the International Commission on Radiological Protection (ICRP) has varied its estimate of the contribution of breast radiation exposure to total body detriment over time, changing the tissue-weighting factor for this organ from 0.05 in 1991 [2] to 0.12 in 2007 [3]. Thus, it is very important to take into account the dose absorbed by the breast during a mammographic exposure, trying to reduce the radiation dose absorbed by patients in every mammographic examination without impairing its diagnostic quality.

This follows the guidelines contained in the European Directive 2013/59/EURATOM issued on 5 December 2013 [4], which gives challenging targets to all stakeholders in terms of justification and optimisation of the procedures using ionising radiation. In any mammographic quality assurance program, special care is required for evaluating and monitoring radiation doses delivered to the breast. In addition, article 58 of the above mentioned directive [4] requires that patients are informed about the risk associated with ionising radiation and that detailed information on the exposure of the patient is included in the report of the radiological procedure.

The average dose absorbed in breast glandular tissue (considered the most sensitive tissue to the radiation-induced carcinogenesis [5]) is a suitable dosimetry quantity [6]. The average glandular dose (*AGD*) is the quantity used to compare dose delivered with different techniques. In 1987, the ICRP recommended the *AGD* as the reference metrics for radiation dose estimation from x-ray mammography, representing the average absorbed dose in the glandular tissue (excluding skin) in a uniformly compressed breast composed of 50% fat and 50% glandular tissue [7]. Therefore, *AGD* has been adopted as dose index in mammography [8, 9] and it is routinely evaluated in quality assurance programs of mammography units.

*AGD* is a fundamental quantity because it is directly related to the risk for patients exposed to ionising radiation by mammography. However, a direct calculation of *AGD* in the breast is not feasible [10]. In fact, the *AGD* depends on the half value layer (HVL) and on the incident air kerma (*k*_*a,i*_) at the upper surface of the breast. These are measurable quantities, but calculations are practically done using tabulated conversion factors, derived from Monte Carlo simulations.

The most used algorithms for *AGD* estimation in mammography are based on the works of Dance et al. [11–13] or of Wu et al. [14, 15]. Following the Dance approach, *AGD* can be calculated by the equation [9]:

1 $$ AGD={k}_{a,i}\cdot g\cdot c\cdot s $$

where *g* is the conversion factor from *k*_*a,i*_ to *AGD* for a standard breast of 50% glandularity; *c* takes into account the differences of breast composition from the standard 50% glandularity (varying in the range 0.885–1.306), and *s* depends on the x-ray spectrum used. *g* and *c* depend on the beam quality (HVL) and on the breast thickness. *c* is also function of the age of the patient (it is different among patients whose ages are in the ranges 40–49 and 50–64 years). All these factors, derived from Monte Carlo simulations, are tabulated and can be found in the above quoted references [11–13].

Following the Wu approach, *AGD* can be calculated by the following equation:

2 $$ AGD={k}_{a,i}\cdot {D}_{gN} $$

where *D*_*gN*_ is the so-called normalised average glandular dose, which depends on HVL, breast thickness, and anode-filter combination. *D*_*gN*_, calculated by Monte Carlo simulations, are tabulated and can be found in literature [14–17].

In this article, a new algorithm for the calculation of the average absorbed breast dose (*2ABD*) is presented. Following this approach, the *2ABD* can be directly calculated for each anode-filter combination based on the *kVp* and *mAs* used and on the breast thickness.

## Methods

### *2ABD*

*2ABD* can be expressed as:

3 $$ 2 ABD=\frac{\int_0^d{k}_{a,i}{e}^{-{\mu}_{en}x} dx}{d}=\frac{k_{a,i}}{\mu_{en}d}\left( 1-{e}^{-{\mu}_{en}d}\right) $$

where *μ*_*en*_ is the energy absorption coefficient in a water-equivalent soft tissue (density 1.0 ± 0.1 g/cm^3^) and *d* is the breast thickness. Note that *μ*_*en*_ depends on *kVp* and anode-filter combination while *k*_*a,i*_ depends on tube-voltage *kVp*, tube current-exposure time product *mAs*, anode-filter combination, *d*, and the distance between the x-ray tube focus and the upper surface of the breast (*FID-d*). *kVp*, *mAs*, anode-filter combination, and *d* are provided by the mammography unit and reported in the DICOM-header file stored for each procedure and *FID* is the (fixed) focus-to-image distance, known for the mammography device used.

### X-ray beam characterisation: evaluation of *k*_*a,i*_

In the range of *kVp* usually employed in mammography (22–34 *kVp*), the incident air kerma on the central axis of the x-ray beam at a distance (*FID-d*) from the tube focus can be linearly related to *kVp*:

4 $$ {k}_{a,i}=\frac{Y_{tb}}{Y_0}\left(\alpha \cdot kVp+\beta \right)\cdot mAs\cdot {\left(\frac{FID}{FID-d}\right)}^2 $$

*Y*_*0*_ and *Y*_*tb*_ are the yields of the reference x-ray tube and the actual x-ray tube. The reference x-ray tube is that used for the experimental measurements needed for *α* and *β* calculation.

In order to determine the parameters *α* and *β*, a set of measurements of *k*_*a,i*_ was done for various *kVp* (range 22–34) and *mAs* (range 10–100) by using a reference x-ray mammography tube (i.e. the mammography unit used for the experimental measurements) whose yield was *Y*_*0*_ for five different anode-filter combinations: *Mo-Mo*, *Mo-Rh* and *Rh-Rh* (Senograph DS General Electric Medical Systems, Waukesha, WI, USA); and *W-Rh* and *W-Ag* (Selenia Dimensions, Hologic, Bedford, MA, USA). *k*_*a,i*_, *kVp* and *mAs* were measured by using a solid-state detector coupled to a multimeter (Piranha®, RTI-Electronics AB, Molndal, Sweden) placed 6 cm from the chest wall edge at the centre of the mammography flat support plate (*d* = 0) with the compression paddle between the x-ray-tube focus and the detector.

Each measurement of *k*_*a,i*_ in the same position, with the same *kVp* and *mAs*, was repeated five times. The average values of *k*_*a,i*_ were fitted (least squares method) to Equation 4 to determine *α* and *β* for each anode-filter combination. The fit uncertainties associated to *α* and *β* were used in the *2ABD* error evaluation.

A comparison of *k*_*a,i*_ calculated by Equation 4 and *k*_*a,i*_ directly measured for different anode-filter combinations and different values of *d* has been done (using four different mammography devices whose yield was *Y*_*tb*_*,* located in different hospitals) to test the accuracy of Equation 4. Note that both *Y*_*0*_ and *Y*_*tb*_ must be calculated at the same value of *kVp* (28 *kVp* in this work).

### Exponential attenuation: evaluation of *μ*_*en*_

The energy absorption coefficient *μ*_*en*_ (*kVp*, anode-filter combination) in a material can be evaluated by the equation:

5 $$ I(x)={I}_0\cdot {e}^{-{\mu}_{en}x} $$

where *x* is the thickness of the attenuating beam-crossed material, *I*_*0*_ is the incident beam intensity, and *I* is the attenuated beam intensity.

A set of experimental measurements was done varying the *kVp* (range 22–34) for each anode-filter combination in order to assess the curves represented by Equation 5. *I*_*0*_ and *I(x)* were evaluated by using a 60-cm^3^ ionisation chamber coupled to a 2026C Radcal Corporation® (Monrovia, CA, USA) electrometer placed under increasing depths *x* (range 0.0–5.5 cm) of solid water (density 1.0 ± 0.1 g/cm^3^). The measured *I*_*0*_ and *I(x)* were fitted to Equation 5 (least squares method) to evaluate *μ*_*en*_.

Once evaluated, *α* and *β* and the energy absorption coefficient *μ*_*en*_ for the five anode-filter combination considered, the *2ABD* was calculated by Eq. 3 for each different mammographic device used.

### Comparison between *AGD* and *2ABD*

Twenty different mammograms were selected from the picture archiving and communication system (PACS) of our university hospital in order to compare *AGD* calculated by the Dance method (Equation 1) [11–13] and the Wu method (Equation 2) [14, 15] to *2ABD* calculated by Equation 3. The three most used *kVp* values were chosen for each anode-filter combination and the most frequent thicknesses were considered for each *kVp*.

*c*, *g*, and *s* used for the *AGD* calculation with the Dance method (Equation 1) are reported in the literature [11–13]; *D*_*gN*_ factors needed for the *AGD* calculation with the Wu method (Equation 2) were also taken from the literature [14–17].

For each mammogram, it was possible to obtain (from the PACS) the anode-filter combination used, the *kVp* and *mAs* set, the breast thickness *d,* and the age of the patients (needed to choose from the Dance’s table the right value of *c* to be used in Equation 1). *Y*_*tb*_ was known from the quality assurance measurements performed on the mammography device under consideration (in this case coincident with *Y*_*0*_) while HVL was measured for each anode-filter combination and *kVp* set in these procedures.

Uncertainties in *AGD* (using Dance or Wu methods) were estimated considering an overall 20% error [18]. The uncertainty in *2ABD* was estimated considering the error propagation for *α*, *β*, *Y*_*0*,_ *Y*_*tb*_, *kVp*, *d*, *FID,* and *μ*_*en*_. In particular, the error of thickness *d* influences both* 2ABD* and *AGD*. The uncertainty in thickness may affect the *AGD* up to 10% [18]. This error component is included in the 20% of total percentage error associated to *AGD* evaluation. For the calculation of *2ABD*, the uncertainty on breast thickness provided by the equipment was considered equal to ± 0.5 cm as reported in the device technical manuals.

The comparison between *AGD* and *2ABD* took into account the overlap between data within their uncertainties.

## Results

**Fig. 1:**
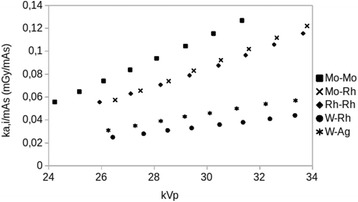
Graphical representation of the measurements done to evaluate *α* and *β* from Equation 4 for five anode-filter combinations

The experimental points used for the calculation of the parameters *α* and *β* by Equation 4 for each anode-filter combination are shown in Fig. 1, where the measured values of *k*_*a,i*_/*mAs* are reported on the *y*-axis as a function of the measured values of *kVp*. *Y*_*tb*_ = *Y*_*0*_ is the yield of the reference x-ray device evaluated for each anode-filter combination at 28 *kVp* and *d* = 0.

The experimental data reported in Fig. 1 were fitted to Equation 4 to calculate the parameters *α* and *β* (Table 1). A linear relationship between *k*_*a,i*_/*mAs* and *kVp* was confirmed in the energy range of interest.

**Table 1:** Yield *Y*_*0*_ (mGy · *mAs*^–1^) of the reference device and *α* (mGy · *mAs*^–1^ · *kVp*^–1^) and *β* (mGy · *mAs*^–1^) for the five anode-filter combinations considered. The average value ± 1 standard deviation of five measurements of *Y*_*0*_ for each anode-filter combination are reported. These measurements were performed at 28 *kVp*

	*Mo-Mo*	*Mo-Rh*	*Rh-Rh*	*W-Rh*	*W-Ag*
*Y*_*0*_	0.0938 ± 0.0008	0.0739 ± 0.0008	0.0707 ± 0.0003	0.031 ± 0.001	0.0391 ± 0.0009
*α*	0.00997 ± 0.00005	0.00892 ± 0.00008	0.0078 ± 0.0002	0.00271 ± 0.00004	0.00371 ± 0.00009
*β*	–0.186 ± 0.001	–0.180 ± 0.002	–0.149 ± 0.006	–0.047 ± 0.001	–0.066 ± 0.003

The results of a set of measurements of *k*_*a,i*_ calculated by Equation 4 are shown in Table 2. The good agreement between the measured and calculated values allows the evaluation of *k*_*a,i*_ by Equation 4 for each mammographic equipment whose yield *Y*_*tb*_ was known. Notice that the determination of *α* and *β* permits the obtaining of *k*_*a,i*_ without directly measuring it.

**Table 2:** Comparison between *k*_*a,i*_ (mGy) measured (*k*_*a,i meas*_) and calculated by Equation 4 (*k*_*a,i calc*_) for different mammography units. *Y*_*tb*_ (mGy · *mAs*^–1^) is the yield of the mammographic device (calculated at 28 *kVp*, different for each anode-filter combination) and *d* (cm) is the breast thickness simulated by different solid-water phantoms. *FID* is the focus-image distance (cm) for the device considered

Device	Anode-filter	*FID*	*Y*_*tb*_	*d*	*kVp*	*mAs*	*k*_*a,i meas*_	*k*_*a,i calc*_	1- (k_a,i calc_/k_a,i meas_)
Fujifilm Amulet 1	Mo-Mo	64	0.094	4	28	20	2.14	2.13	–0.01
Fujifilm Amulet 2	Mo-Mo	64	0.090	6	28	20	2.23	2.16	–0.03
Fujifilm Amulet 1	Mo-Rh	64	0.075	2	28	20	1.62	1.61	–0.01
Fujifilm Amulet 2	Mo-Rh	64	0.079	4	28	20	1.80	1.91	–0.06
GE Senographe DS	Rh-Rh	63.5	0.072	5	29	50	4.80	4.65	0.03
GE Senographe DS	Rh-Rh	63.5	0.072	3	27	40	2.83	2.67	0.06
Fujifilm Amulet 1	W-Rh	64	0.031	4	28	20	0.71	0.79	0.10
Fujifilm Amulet 2	W-Rh	64	0.030	6	28	20	0.71	0.81	0.11
Giotto class	W-Ag	67	0.0394	4	26	93	3.36	3.43	0.02
Giotto class	W-Ag	67	0.0394	6	29	93	5.06	4.98	–0.02

**Fig. 2:**
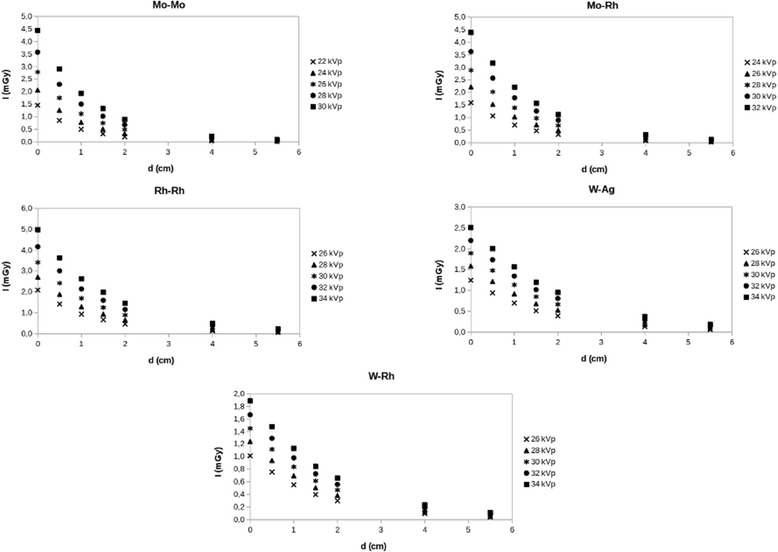
Experimental measurements of *I(x)* for different anode-filter combinations and different thickness of water. The experimental points represent the average values of four measurements of *I(x)*

The graphical representation of Equation 5 for each anode-filter combination is shown in Fig. 2. The points represent the average values of four measurements of *I(x)* done by using a 60-cm^3^ ionisation chamber placed at different depths in a solid-water phantom. Data showed the expected exponential decay trend, thus the experimental points were fitted by Equation 5 to evaluate the energy absorption coefficient *μ*_*en*_. The results of the fit are reported in Table 3 for each anode-filter combination.

**Table 3:** *I*_*0*_ (mGy) and *μ*_*en*_ (cm^–1^) for each anode-filter combination for different *kVp*. The coefficient R^2^ > 0.99 for each anode-filter combination states the goodness-of-fit of the experimental measurements (Fig. 2) to Equation 5. *mAs* value was set to 40 for each anode-filter combination

	***Mo-Mo***				
*kVp*	22	24	26	28	30
*I*_*0*_	1.45 ± 0.04	2.05 ± 0.05	2.77 ± 0.06	3.55 ± 0.08	4.41 ± 0.09
*μ*_*en*_	1.04 ± 0.05	0.95 ± 0.04	0.89 ± 0.04	0.84 ± 0.04	0.81 ± 0.03
	***Mo-Rh***				
*kVp*	24	26	28	30	32
*I*_*0*_	1.59 ± 0.03	2.22 ± 0.03	2.88 ± 0.03	3.62 ± 0.04	4.39 ± 0.06
*μ*_*en*_	0.80 ± 0.03	0.75 ± 0.02	0.72 ± 0.02	0.70 ± 0.02	0.68 ± 0.02
	***Rh-Rh***				
*kVp*	26	28	30	32	34
*I*_*0*_	2.08 ± 0.06	2.69 ± 0.07	3.39 ± 0.09	4.1 ± 0.1	4.9 ± 0.8
*μ*_*en*_	0.76 ± 0.04	0.71 ± 0.04	0.67 ± 0.04	0.64 ± 0.03	0.61 ± 0.03
	***W-Rh***				
*kVp*	26	28	30	32	34
*I*_*0*_	1.02 ± 0.02	1.25 ± 0.02	1.46 ± 0.02	1.68 ± 0.02	1.90 ± 0.03
*μ*_*en*_	0.61 ± 0.02	0.59 ± 0.02	0.56 ± 0.02	0.54 ± 0.02	0.53 ± 0.02
	***W-Ag***				
*kVp*	26	28	30	32	34
*I*_*0*_	1.25 ± 0.02	1.59 ± 0.02	1.90 ± 0.03	2.21 ± 0.03	2.52 ± 0.04
*μ*_*en*_	0.58 ± 0.02	0.55 ± 0.02	0.52 ± 0.03	0.50 ± 0.02	0.48 ± 0.02

As reported in Table 3, *μ*_*en*_ depends weakly on the *kVp* set in the considered energy range. Therefore, an average value of *μ*_*en*_ was chosen for each anode-filter combination (Table 4). For a given anode-filter combination, the difference between *2ABD* derived from Equation 3 considering the average value of *μ*_*en*_ and the *μ*_*en*_(*kVp*) value (i.e. the *μ*_*en*_ obtained for a specific *kVp* value) was negligible.

**Table 4:** Average values of energy absorption coefficients *μ*_*en*_ (cm^–1^) for each anode-filter combination. These values are used in Equation 3

Anode-filter	*μ*_*en*_
*Mo-Mo*	0.91 ± 0.04
*Mo-Rh*	0.73 ± 0.02
*Rh-Rh*	0.68 ± 0.04
*W-Rh*	0.57 ± 0.02
*W-Ag*	0.53 ± 0.02

The comparison between *AGD*, calculated by the Dance method (Equation 1), *AGD* calculated by the Wu method (Equation 2) and *2ABD* calculated by Equation 3 are shown in Table 5 for 20 different mammograms. The characteristics of the patients and the data of the exams, needed for the calculation of *AGD* and *2ABD* are also reported in Table 5.

**Table 5:** Characteristics of patients and data of the exams used for the comparison between *AGD* (mGy) calculated by the Dance method (by Geeraert et al. 1), *AGD* (mGy) calculated by the Wu method (by Geeraert et al. 2) and *2ABD* (mGy) (by Geeraert et al. 3). *c*, *g* and *s* used for the calculation of *AGD* by by Geeraert et al. 1 were found in [11–13]; *D*_*gN*_ used in by Geeraert et al. 2 were found in [17]. *d* is expressed in cm; HVL in mmAl. The glandularity (%) of the breast has been evaluated following Dance et al

Patient no.	Age (years)	Glandularity (%)	Anode-filter	*d*	*kVp*	*mAs*	HVL	*AGD* (Dance)	AGD (Wu, Boone)	*2ABD*
1	61	100	Mo-Mo	2	25	32	0.30	0.7 ± 0.1	0.6 ± 0.1	1.0 ± 0.3
2	55	72	Mo-Mo	3	26	36	0.30	0.7 ± 0.1	0.6 ± 0.1	1.0 ± 0.2
3	43	91	Mo-Mo	2.4	27	18	0.31	0.45 ± 0.09	0.40 ± 0.08	0.6 ± 0.2
4	60	72	Mo-Rh	3	26	43	0.42	0.8 ± 0.2	0.7 ± 0.1	0.9 ± 0.3
5	48	65	Mo-Rh	4	27	50	0.43	0.9 ± 0.2	0.8 ± 0.2	1.1 ± 0.2
6	51	50	Mo-Rh	4	28	54	0.44	1.2 ± 0.2	1.1 ± 0.2	1.3 ± 0.3
7	63	33	Rh-Rh	5	28	48	0.45	1.0 ± 0.2	1.0 ± 0.2	1.1 ± 0.3
8	59	33	Rh-Rh	5	29	58	0.47	1.4 ± 0.3	1.4 ± 0.3	1.4 ± 0.4
9	45	35	Rh-Rh	6	29	73	0.47	1.5 ± 0.3	1.5 ± 0.3	1.6 ± 0.4
10	57	21	Rh-Rh	6	30	63	0.48	1.5 ± 0.3	1.6 ± 0.3	1.5 ± 0.4
11	60	12	Rh-Rh	7	30	75	0.48	1.7 ± 0.3	1.7 ± 0.3	1.6 ± 0.4
12	55	42	W-Rh	4.5	28	72	0.54	0.8 ± 0.2	0.7 ± 0.1	0.8 ± 0.2
13	43	49	W-Rh	5	29	95	0.54	1.0 ± 0.2	0.9 ± 0.2	1.1 ± 0.2
14	46	42	W-Rh	5.5	30	99	0.56	1.1 ± 0.2	1.1 ± 0.2	1.2 ± 0.2
15	46	49	W-Rh	5	29	89	0.54	0.9 ± 0.2	0.9 ± 0.2	1.0 ± 0.2
16	42	65	W-Rh	4	28	103	0.54	1.1 ± 0.2	1.0 ± 0.2	1.3 ± 0.3
17	52	12	W-Ag	7	30	114	0.62	1.7 ± 0.3	1.8 ± 0.4	1.6 ± 0.3
18	47	24	W-Ag	7	30	68	0.62	1.0 ± 0.2	1.0 ± 0.2	1.0 ± 0.2
19	40	24	W-Ag	7	30	77	0.62	1.1 ± 0.2	1.1 ± 0.2	1.1 ± 0.2
20	52	12	W-Ag	7	30	105	0.62	1.6 ± 0.3	1.6 ± 0.3	1.5 ± 0.3

The uncertainty on *2ABD* considering each error contribution varied in the range 17-33% (Table 5).

The three methods appear to be consistent within the uncertainties. A good agreement can be observed among data in Table 5 for each specific patient.

## Discussion

*2ABD*, defined as the average value of energy absorbed per unit mass in the breast, is a suitable, easily measurable physical quantity, related to the patient exposure in mammographic procedures. The method presented in this paper requires the knowledge of *kVp*, *mAs*, breast thickness *d*, and anode-filter combination. All these parameters are set on the mammographic device and they are reported also in the DICOM-header file associated to each image. *Y*_*tb*_ can be periodically calculated for each device and anode-filter combination, during the quality controls; *FID* is a characteristic of the device. Thus, all these parameters are available and could be employed to implement an automatic calculation of *2ABD* for each mammographic procedure. Then, *2ABD* can be recorded in the report of each exam.

Different anode-filter combinations were characterised in order to evaluate *k*_*a,i*_ by Equation 4 without needing to measure it directly in each procedure. This could avoid inserting expensive measurements systems (which must be periodically controlled and calibrated) in the different mammographic devices.

*AGD* calculated by the Dance method (Equation 1) or the Wu method (Equation 2) represents the reference standard in mammography quality assurance programs. Even though the *AGD* does not take into account the fundamental individual radiobiological factors (i.e. radiosensitivity and radiosusceptivity), it has been directly related to the risk of carcinogenesis due to the exposition of the breast tissue to x-ray [5]. Thus, it can be considered as the dose index to be written in the report of the mammographic procedure, as explicitly requested by the 2013 European Council Directive [4]. According to Dance [10], *AGD* cannot be directly measured. It needs a dedicated detector for the *k*_*a,i*_ evaluation. It is also a function of the HVL, which strictly depends on *kVp* and anode-filter combination, as well as on other non-easily measurable parameters such as breast density. For these reasons, *AGD* can be evaluated in breast phantoms, but its calculation for individual patients is inaccurate [19]. As a consequence, the possibility to directly communicate the *AGD* in the mammographic report is questionable. Conversely, the new proposed dose index *2ABD* can be easily calculated from parameters set for each mammogram and stored in the DICOM-header of each patient image, avoiding direct *k*_*a,i*_ measurements. Thus, *2ABD* could be easily automatically included in every mammographic report, saving time and facilitating quality assurance.

A comparison between *AGD*, calculated by Equations 1 and 2, and *2ABD*, calculated by Equation 3 (with *k*_*a,i*_ evaluated by Equation 4), was done for 20 procedures selected for different anode-filter combinations and different breast thickness.

Differently from Dance et al. [10–13] or Wu et al. [14, 15], *2ABD* does not take into consideration differences in breast density (whose range is 0.93–1.04 g/cm^3^ [6]). Dance relates breast density to the age of the patient (he considers two ranges of age, 40–49 years or 50–64 years) and to the breast thickness [13]. As reported by Geeraert et al. [20], the evaluation of breast density is a critical factor affecting *AGD* accuracy. Based on these considerations, the opportunity to consider the breast density in the routinely breast dose calculation for each patient appears to be questionable and should be further discussed.

Anyway, despite the non-consideration of breast density by our *2ABD* method, Table 5 does not show evident discrepancy between *AGD* and *2ABD* values, although a different set of radiation exposure and patient-related parameters was involved in each mammographic procedure. The results of the comparison between *2ABD* and *AGD* evaluated by either Dance or Wu methods appear to be consistent within the uncertainties.

Thus, *2ABD* could work as surrogate of *AGD* and could be taken into consideration as a proxy of *AGD*, to be provided for each mammographic procedure, having the *2ABD* calculation algorithm implemented in the software of the mammographic device.

## Abbreviations

2ABD: Average absorbed breast dose
AGD: Average glandular dose
DICOM: Digital imaging and communications in medicine
FID: Focus-to-image
HVL: Half value layer
ICRP: International Commission on Radiological Protection
PACS: Picture archiving and communication system
